# Contralateral Arm Pain as a Sign of Distress Regarding Symptoms

**DOI:** 10.1177/15589447231216145

**Published:** 2023-12-11

**Authors:** Floor A. Davids, Jose C. Padilla, David Ring, Gregg A. Vagner, Lee M. Reichel, Sina Ramtin

**Affiliations:** 1 The University of Texas at Austin, USA

**Keywords:** upper extremity, non-trauma, pain, capability, unhelpful thoughts, distress

## Abstract

**Background:**

Pain intensity and magnitude of incapability are associated with common unhelpful thoughts about symptoms such as catastrophic thinking and kinesiophobia. To determine whether reports of pain in the upper limb contralateral to a non-trauma condition were associated with unhelpful thoughts, we measured the relationship between pain intensity in the opposite limb and levels of unhelpful thinking.

**Methods:**

In a cross-sectional study, 152 new and return patients seeking care of an upper-limb musculoskeletal condition completed measures of upper-extremity-specific magnitude of capability, pain intensity of the involved and contralateral arms, unhelpful thoughts regarding symptoms, symptoms of distress regarding symptoms, and general symptoms of depression. Factors associated with contralateral and ipsilateral pain intensity and upper-extremity-specific magnitude of capability were assessed using multivariable statistics.

**Results:**

In bivariate analysis, contralateral arm pain was associated with symptoms of distress regarding pain, but not in multivariable analysis. Accounting for potential confounding in negative binominal regression analysis, greater pain intensity of the affected side was independently associated with greater feelings of distress regarding symptoms and no prior surgery. Greater upper-extremity-specific capability was independently associated with less distress regarding symptoms, married/partnered, men, and no prior surgery.

**Conclusions:**

The observation that greater pain intensity in the opposite arm was associated with greater distress regarding symptoms suggests that, in combination with other verbal and non-verbal signs of distress, patient concerns about pain in the contralateral limb can help direct patients and surgeons to evidence-based care strategies for alleviating stress regarding symptoms.

## Introduction

### Background

People seeking specialty care often provide verbal^1^ and non-verbal^2^ clues that they are experiencing unhelpful thoughts or feelings of distress regarding sensations. A study using facial recognition of emotions documented that clinicians register these clues, perhaps in part subconsciously.^3^ These thoughts and feelings about symptoms are a good target for efforts to improve health because they are associated with greater pain intensity and lower magnitude of capability.^4-8^

### Rationale

People with unilateral non-traumatic upper-extremity conditions may adjust their use of the contralateral arm. Greater use of the contralateral arm and any associated pains could be considered healthy exercise.^9^ When use is associated with pain, some patients may interpret the increased use as “overuse” or “overcompensation.” Such interpretations seem to be a product of the human mind’s tendency to interpret painful activity as activity that will make the problem worse.^4,10^ Expressions of concern regarding discomfort in the contralateral arm might therefore represent a clue that the patient is experiencing unhelpful thoughts (such as catastrophic thinking or kinesiophobia) or feelings of worry or despair regarding symptoms. If expressions of concern about discomfort in the contralateral limb are associated with unhelpful thinking, clinicians could use them as an indicator—or awareness tool—for common unhealthy misconceptions about pain. Enhanced awareness of the words and movements characteristic of unhelpful thinking or distress regarding symptoms has the potential to bolster strategies for anticipating, identifying, and gently reorienting common misinterpretations of symptoms and associated feelings of worry or despair.

We asked, “Are there any factors, including unhelpful thoughts or feelings of distress, associated with contralateral arm pain intensity among people with a unilateral non-trauma upper condition?” We also asked, “Are there any factors associated with ipsilateral pain intensity and magnitude of capability among people with a unilateral non-trauma upper condition?”

## Methods

### Study Design and Setting

Between December 2021 and February 2022, English-speaking adult (age 18 years and older) patients with a unilateral non-trauma upper condition presenting to a musculoskeletal specialist in one of several outpatient offices in a metropolitan area in the United States were invited to participate in this cross-sectional study by an independent research assistant. The protocol was approved by our institutional review board. Verbal informed consent was obtained by the research assistant. Completion of the survey was accepted in lieu of written informed consent. Patients were excluded if they were English illiterate, if they had bilateral symptoms, or if they had notable cognitive deficiencies.

Patients were asked to complete measures of pain intensity, magnitude of capability, symptoms of depression, unhelpful thoughts and distress about symptoms,^10^ and basic demographics. Surveys were completed on a tablet using Health Insurance Portability and Accountability Act (HIPAA)-compliant survey software REDcap (Research Electronic Data Capture, Vanderbilt, Tennessee).

### Participants

A total of 161 patients started the survey. Nine responses that were less than 50% complete were omitted, leaving 152 participants for analysis. The median (interquartile range [IQR]) age was 57 years (45 to 67). Forty-three percent (n = 65) were men (Table 1). Diagnoses were not tracked and were representative of the most common conditions treated by a hand and upper-extremity surgeon.

**Table 1.** Demographics.

*N*	**152**
Gender	
Female	57% (87)
Male	43% (65)
Race/ethnicity	
White	68% (104)
Latino/Hispanic	16% (25)
Asian	6.6% (10)
African-American	4.6% (7)
Other	4.0% (6)
Marital status	
Married/partner	68% (103)
Single	18% (27)
Divorced/separated	7.9% (12)
Widowed	5.9% (9)
Other	0.66% (1)
Highest level of education	
Highschool or less	5.9% (9)
Some college	25% (38)
College graduate	40% (61)
Masters degree	21% (32)
PhD or professional degree	7.9% (12)
Work status	
Full-time employed	53% (81)
Parttime employed	8.6% (13)
Retired	29% (44)
Disabled	1.3% (2)
Unemployed	4.6% (7)
Student	3.3% (5)
Insurance type	
Medicare	32% (49)
Medicaid	1.3% (2)
Private	64% (98)
Workers’ compensation	2.0% (3)
Dominant hand	
Left-handed	10% (14)
Right-handed	90% (126)
Affected hand	
Left hand	41% (58)
Right hand	59% (82)
Affected hand same as dominant hand	
No	39% (60)
Yes	61% (92)
Prior surgery	
No	83% (116)
Yes	17% (24)
Continuous variables^a^	
Age	57 (45-67)
PROMIS Depression	48 (SD 7.6)
Duration of symptoms (weeks)	12 (5.5-49)
Unhelpful thoughts regarding symptoms	3.0 (2.0-5.0)
Distress regarding symptoms	2.0 (0-5.0)

*Note.* PROMIS = Patient-Reported Outcomes Measurement Information System.

a Values are displayed as median with interquartile range for continuous non-parametric variables, as mean with SD for continuous variables with normal distribution, and as number with percentage for categorial variables.

### Measurements

We measured pain intensity using the Numeric Rating Scale (NRS) for Pain, an 11-point numeric scale between 0 (no pain) and 10 (the worst pain imaginable).^11^ Pain in the affected (ipsilateral) and unaffected (contralateral) upper extremity was measured separately.

We measured upper-extremity-specific magnitude of incapability using the Patient-Reported Outcomes Measurement Information System (PROMIS) Upper Extremity (UE) Physical Function (PF) Computer Adaptive Test (CAT).^12^ All PROMIS CAT scores are reported as a t-score with a score of 50 representing the mean score for the general population living in the United States. Ten points above or below the mean score represents one SD variation. Higher scores indicate greater capability.

We used PROMIS Depression CAT to quantify symptoms of depression, self-reported negative mood (sadness and guilt), views of self (self-criticism and worthlessness), social cognition (loneliness and interpersonal alienation), and decreased positive affect and engagement (loss of interest, meaning, and purpose).^13^ Higher scores indicate more symptoms of depression.

We measured unhelpful thoughts and feelings of distress, each using three questions selected in a prior factor analysis.^10^ Each question was rated on a 5-point scale with each point labeled: 1 (Strongly disagree), 2 (Disagree), 3 (Neutral), 4 (Agree), and 5 (Strongly agree).

### Statistical Analysis

Descriptive statistics were performed for all demographic variables. Continuous variables with parametric distribution were described as mean with SD, and continuous variables with non-normal distribution were described as median with IQR. Categorical data were described and as a number with percentage.

We performed a bivariate analysis to identify factors associated with pain intensity in the contralateral and affected extremity and with magnitude of capability (PROMIS UE PF CAT). The relationship of explanatory variables with pain intensity was tested using Mann-Whitney U tests and Kruskal-Wallis H tests for non-parametric continuous data and the Spearman correlation coefficient for continuous explanatory variables. Variables with *P* values < .10 in bivariable analysis (Table 2) were moved to multivariable analysis. Collinearity between variables was checked using bivariate analysis between two variables that can potentially and logically be related, and employment status and insurance type were found to be colinear. We selected the mental health measure with the strongest correlation in bivariate analysis for inclusion in the multivariable model because our past experience suggests that the mental health measures have notable interrelationships that can alter multivariable models even if they do not meet traditional thresholds for collinearity. A negative binomial regression model was used to identify factors associated with pain intensity in both the contralateral and the affected extremity. A linear regression model was used to identify factors associated with the magnitude of capability. All variables with a *P* value < .05 were considered statistically significant.

**Table 2.** Bivariate Analysis of Factors Associated With Pain in the Contralateral Arm, Pain in the Ipsilateral Arm, and Magnitude of Capability.

	**Pain in contralateral arm**		**Pain in ipsilateral arm**		**Magnitude of capability**	
Variable	Median (IQR)	*P* value	Median (IQR)	*P* value	Median (IQR)	*P* value
Gender		.62		.71		**.0036**
Female	0 (0-1)		4 (2-6)		40 (35-46)	
Male	0 (0-2)		4 (2-6)		46 (37-51)	
Race/ethnicity		**.024**		**.011**		.11
Other	0 (0-3)		5 (3-6)		40 (31-46)	
White	0 (0-1)		3 (1-5)		43 (36-50)	
Marital status		.84		.53		**<.001**
Divorced/separated/single	0 (0-1)		4 (2-6)		37 (31-42)	
Married/partner	0 (0-1)		4 (2-6)		45 (37-51)	
Highest level of education		.23		**.024**		.13
Some college or less	0 (0-3)		5 (2-7)		40 (36-46)	
College graduate	0 (0-1)		4 (2-5)		42 (34-49)	
Masters or PhD degree	0 (0-1)		3 (1-5)		45 (37-50)	
Work status		.43		.16		.082
Unemployed/student/retired	0 (0-1)		3 (2-5)		41 (31-46)	
Employed	0 (0-2)		4 (2-6)		43 (37-49)	
Insurance type		.76		.25		.076
Non-private	0 (0-1)		3 (1-5)		40 (35-46)	
Private	0 (0-1)		4 (2-6)		43 (36-49)	
Prior surgery		.61		**<.001**		**.016**
No	0 (0-1)		4 (2-6)		43 (36-50)	
Yes	0 (0-1)		2 (1-3)		37 (34-42)	
Affected hand same as dominant hand		**.016**		.82		**.029**
No	0 (0-0)		4 (2-6)		44 (37-51)	
Yes	0 (0-2)		4 (2-6)		41 (34-47)	
**Continuous variables**	Spearman correlation coefficient	*P* value	Spearman correlation coefficient	*P* value	Spearman correlation coefficient	*P* value
Age	.026	.76	−.059	.49	−.11	.18
PROMIS Depression	.082	.34	.30	**<.001**	−.12	.15
Duration of symptoms (weeks)	.081	.34	−.029	.74	.042	.62
Unhelpful thoughts regarding symptoms	.13	.12	.20	**.017**	−.12	.15
Distress regarding symptoms	.28	**<.001**	.52	**<.001**	−.17	**.042**

*Note.* Boldface values indicate statistical significance, *P* < .05; all response variables were reported as median (IQR) due to non-parametrical distribution. The median is the middle number of a distribution and so does not give any information on the difference between groups because of the notable skew in the distribution. The IQR is a better indication of the differences in distribution noted to be significant with Kruskal-Wallis or Mann-Whitney tests. IQR = interquartile range; PROMIS = Patient-Reported Outcomes Measurement Information System.

## Results

### Contralateral Arm Pain Intensity

Accounting for potential confounding among factors with *P* < .10 in bivariate analysis (including non-White race/non-Hispanic ethnicity, involvement of the dominant hand, and distress regarding symptoms), no factors were associated with contralateral arm pain intensity (all *P* values were more than .05) (Tables 2 and 3).

**Table 3.** Negative Binomial Regression of Factors Associated With Pain in the Contralateral Arm.

Variable	Regression coefficient (95% confidence interval)	Standard error	*P* value
Race/ethnicity			
Other	Reference value		
White	−0.55 (−1.3 to 0.17)	0.37	.14
Affected hand same as dominant hand			
No	Reference value		
Yes	0.41 (−0.28 to 1.1)	0.35	.25
Distress regarding symptoms	0.11 (−0.027 to 0.24)	0.068	.12

### Ipsilateral Arm Pain Intensity

In negative binomial regression analysis accounting for potential confounders such as PROMIS Depression and unhelpful thoughts regarding symptoms, greater pain intensity in the affected arm was associated with greater symptoms of distress regarding symptoms (regression coefficient [RC] = 0.084; 95% CI: 0.048-0.12; *P* < .001). Lower pain intensity in the affected arm was associated with prior surgery (RC = −0.57; 95% CI:−0.86 to −0.28; *P* < 0.001) (Table 4).

**Table 4.** Negative Binomial Regression of Factors Associated With Pain in the Ipsilateral Arm.

Variable	Regression coefficient (95% confidence interval)	Standard error	*P* value
Race/ethnicity			
Other	Reference value		
White	−0.091 (−0.28 to 0.095)	0.095	.34
Highest level of education			
Some college or less	Reference value		
College graduate	−0.12 (−0.32 to 0.082)	0.10	.24
Masters or PhD degree	−0.18 (−0.42 to 0.062)	0.12	.15
Prior surgery			
No	Reference value		
Yes	−0.57 (−0.86 to −0.28)	0.15	**<.001**
PROMIS Depression	0.009 (−0.005 to 0.022)	0.0067	.20
Unhelpful thoughts regarding symptoms	−0.015 (−0.060 to 0.029)	0.023	.50
Distress regarding symptoms	0.084 (0.048 to 0.12)	0.019	**<.001**

*Note.* Boldface values indicate statistical significance, *P* < .05. PROMIS = Patient-Reported Outcomes Measurement Information System.

### Magnitude of Capability

In a linear regression analysis, greater upper-extremity-specific capability was associated with men (RC = 4.4; 95% CI: 1.2-7.5; *P* = .007), being married/partnered (RC = 4.5; 95% CI: 1.1-7.9; *P* = .010). Lower capability of the upper extremity was associated with greater distress regarding symptoms (RC = −0.63; 95% CI: −1.2 to −0.086; *P* = .023) and prior surgery (RC = −5.3; 95% CI: −9.4 to 1.1; *P* = .013) (Table 5).

**Table 5.** Linear Regression of Factors Associated With Magnitude of Capability.

Variable	Regression coefficient (95% confidence interval)	Standard error	*P* value
Gender			
Female	Reference value		
Male	4.4 (1.2 to 7.5)	1.6	**.007**
Marital status			
Divorced/separated/single	Reference value		
Married/partner	4.5 (1.1 to 7.9)	1.7	**.010**
Insurance type			
Non-private	Reference value		
Private	1.7 (−1.7 to 5.0)	1.7	.33
Prior surgery			
No	Reference value		
Yes	−5.3 (−9.4 to −1.1)	2.1	**.013**
Affected hand same as dominant hand			
No	Reference value		
Yes	−2.3 (−5.5 to 0.83)	1.6	.15
Distress regarding symptoms	−0.63 (−1.2 to −0.086)	0.27	**.023**

*Note.* Boldface values indicate statistical significance, *P* < .05.

## Discussion

There is growing interest in the relationship between mindset and circumstances and musculoskeletal comfort and capability (biopsychosocial paradigm).^14-17^ Unhelpful thinking (misconceptions) and distress regarding symptoms account for a notable amount of the variation in pain intensity and magnitude of capability among people presenting for care of a musculoskeletal condition.^18-22^ There is evidence that patients provide verbal and non-verbal clues that reflect unhelpful thinking and feelings of distress.^1,2^ Patients seeking musculoskeletal specialty care for a non-traumatic upper-extremity problem often report increased use of the contralateral limb as potentially harmful. This is a common misconception that might be a clue for the clinician to reorient unhelpful thinking. Our study found that greater contralateral arm pain intensity was associated with distress regarding symptoms in bivariate, but not multivariable analysis.

The current study has some limitations. First, the study population was largely White and educated people living in an urban area which might make our findings less applicable to other populations. However, our analysis addresses associations with human mental health traits rather than absolute rates, and associations are likely consistent across different study populations with adequate variation in mental health measures. Second, we enrolled patients with a variety of non-traumatic upper-extremity conditions. Because the surveys were completed before the visit, we did not track diagnoses. The distribution of diagnoses should be typical of hand specialty care. The results might vary with specific types of pathology, but that seems unlikely, and there are advantages to having a typical cross-section of upper-extremity conditions represented. Third, there was a small number of incomplete surveys related to allowing people to use their own device, which we no longer allow. We do not think this would have much influence on the results. Fourth, we used a subset of questions from validated surveys based on a prior study demonstrating that they are representative of the full questionnaire.^10^ In our opinion, this is sufficient validation and an important step toward using a small number of relevant questions with patients at the point of care.

The finding that greater contralateral arm pain intensity was related to greater distress regarding symptoms in bivariate, but not in multivariable analysis, suggests that reports of pain in the contralateral upper limb might be considered a minor clue that there are opportunities for improved health by addressing unhelpful thoughts and feelings of distress regarding symptoms, but further investigation is merited. It is not clear why distress was not retained in multivariable analysis and where the confounding might arise. For the time being, based on the results of other studies on this topic, combined with the relationship in the bivariate analysis, we feel that it might be useful for clinicians to be attuned to reports of contralateral upper-limb pain, become aware of the association with less healthy mindsets, and strategize alleviation of feelings of distress.^20,21^

The finding that greater ipsilateral arm pain intensity and magnitude of incapability are associated with greater symptoms of distress regarding symptoms is consistent with extensive previous evidence that variations in comfort and capability have notable associations with unhelpful thinking and symptoms of distress, more so than variations in pathophysiology.^6,7,23-26^ Distress regarding symptoms is common among patients with unilateral non-traumatic upper-extremity conditions and can be anticipated by clinicians. Specialist ability to identify and address misconceptions and distress regarding symptoms can help alleviate symptoms through reorientation of misconceptions and alleviation of feelings of distress. The finding relationships with prior surgery might correspond with pathology severity, mindset factors, or health care behaviors among other things.^23-28^

The findings that, among patients with a unilateral non-traumatic upper condition, expressions of pain in the contralateral arm correlated with distress regarding symptoms in bivariate, but not in multivariable analysis, suggests a potential association that merits additional testing. If even a limited relationship can be confirmed, reports of pain in the contralateral limb might be considered a useful verbal indicator of distress regarding symptoms. This clue could then be used by surgeons as a cue to diagnose and treat symptoms of distress and strategize their alleviation.
